# Magnetic force-based cell manipulation for *in vitro* tissue engineering

**DOI:** 10.1063/5.0138732

**Published:** 2023-09-19

**Authors:** Huiqian Hu, L. Krishaa, Eliza Li Shan Fong

**Affiliations:** 1 Department of Biomedical Engineering, National University of Singapore, Singapore, Singapore; 2 The N.1 Institute for Health, National University of Singapore, Singapore, Singapore; 3 Cancer Science Institute of Singapore, National University of Singapore, Singapore, Singapore

## Abstract

Cell manipulation techniques such as those based on three-dimensional (3D) bioprinting and microfluidic systems have recently been developed to reconstruct complex 3D tissue structures *in vitro*. Compared to these technologies, magnetic force-based cell manipulation is a simpler, scaffold- and label-free method that minimally affects cell viability and can rapidly manipulate cells into 3D tissue constructs. As such, there is increasing interest in leveraging this technology for cell assembly in tissue engineering. Cell manipulation using magnetic forces primarily involves two key approaches. The first method, positive magnetophoresis, uses magnetic nanoparticles (MNPs) which are either attached to the cell surface or integrated within the cell. These MNPs enable the deliberate positioning of cells into designated configurations when an external magnetic field is applied. The second method, known as negative magnetophoresis, manipulates diamagnetic entities, such as cells, in a paramagnetic environment using an external magnetic field. Unlike the first method, this technique does not require the use of MNPs for cell manipulation. Instead, it leverages the magnetic field and the motion of paramagnetic agents like paramagnetic salts (Gadobutrol, MnCl_2_, etc.) to propel cells toward the field minimum, resulting in the assembly of cells into the desired geometrical arrangement. In this Review, we will first describe the major approaches used to assemble cells *in vitro*—3D bioprinting and microfluidics-based platforms—and then discuss the use of magnetic forces for cell manipulation. Finally, we will highlight recent research in which these magnetic force-based approaches have been applied and outline challenges to mature this technology for *in vitro* tissue engineering.

## I. INTRODUCTION

Living cells can typically be manipulated to enable single-molecule or single-cell detection with the use of optical tweezers^1^ or to enable cell separation or biophysical cellular characterization with the use of microfluidics-based systems.^2^ In the field of tissue engineering, living cells are manipulated to enable the formation of structured tissues or organs, such as with the use of three-dimensional (3D) bioprinting technologies,^3^ which recapitulate tissue geometry and function *in vitro*. In recent years, as a result of advances in spatial transcriptomics technologies, we now have an improved understanding of how gene activity orchestrates complex cellular assemblies in multicellular organs^4^ and how tissue organization can affect the cellular phenotype.^5^ In normal tissues, tissue architecture is essential as it maintains mechanical properties and provides specific microenvironments to influence cell phenotype and state.^6^ In cancer, stromal cells (e.g., immune cells, fibroblasts) are uniquely distributed with respect to cancer cells and can influence the cancer cell phenotype and drug response.^5,7^

To enable the assembly of multicellular tissues and organs, two key approaches have emerged over the years, namely, scaffold-based and scaffold-free methods. In scaffold-based approaches, microfluidic systems and 3D bioprinting/printing technologies have emerged as highly useful tools for tissue reconstruction *in vitro*. Scaffolds are typically used to provide *in vivo*-like cell–matrix interactions to support specific cell states and spatially organize different cell populations in three dimensions.^8^ At the micro-level, microfluidic-based systems not only enable perfusion to support large multicellular constructs,^9^ but these systems can also support consistent miniaturization, integration, automation, and parallelization, which allow integration with imaging systems and analytical tools like Raman or mass spectrometry.^10^ Furthermore, microfluidic approaches have been successfully leveraged to design organ-on-a-chip (OOC) technologies to recapitulate the architecture of organs to study disease progression and drug response.^11^ Recently, in a landmark study, interdependent organ functions were recapitulated through the development of a tissue-on-chip system, in which matured human heart, liver, bone, and skin tissue niches were connected by recirculating vascular flow.^12^ However, microfluidics technologies also present several disadvantages. The complex fabrication process makes it very challenging for researchers with little to no microfabrication background to build microfluidic-based platforms. Moreover, even if the chip were to be successfully fabricated, optimization of flow conditions like droplet size and flow rate is time-consuming and difficult to keep consistent. At the macro-level, 3D bioprinting technologies leverage bioinks composed of cells and biomaterials originating from extracellular matrix (ECM) components or synthetic materials to recapitulate the structure of complex tissues. The use of natural or synthetic materials as part of bioinks present unique challenges; ECM-based biomaterials, such as collagen and hyaluronan, offer excellent biocompatibility for many tissue engineering applications, but the mechanical properties of these natural biomaterials are often poor even after cross-linking. This makes it challenging to use these materials to reconstruct tissues that need to withstand high mechanical loading.^13^ Synthetic biomaterials such as functionalized poly(ethylene) glycol enable tunability of physical and biochemical properties. However, some of these materials may be associated with poor biocompatibility, cytotoxic degradation products, and inadequate bioactivity.^14^ Furthermore, the bioprinting process is time-consuming and may present high costs.^15^

**FIG. 1.**
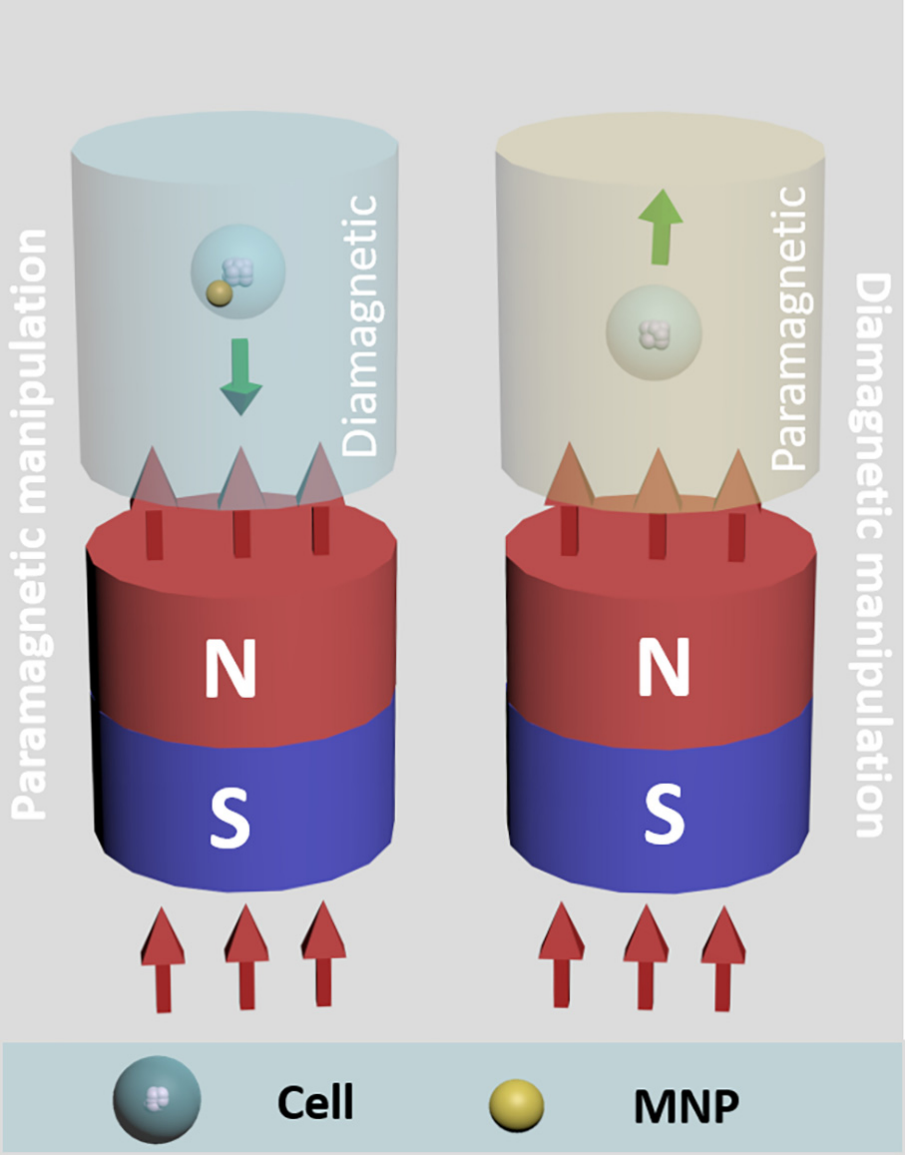
Two main categories of magnetic manipulation. For paramagnetic manipulation (left panel), magnetic nanoparticles (MNPs) are internalized by cells in a diamagnetic medium, cells are then driven by the magnetic field to the field maximum through attractive forces. For diamagnetic manipulation (right panel), cells are suspended in a paramagnetic medium; they are then driven by synergistic repulsion from the magnetic field and the attraction-induced movement of paramagnetic salts, and move to the field minimum.

As an alternative approach to microfluidic systems and 3D bioprinting/printing technologies, magnetic force-based technologies have emerged as a means to generate multicellular tissue constructs at low cost and with high throughput. Magnetic force-based methods for cell manipulation fall into two main categories (Fig. 1). The first commonly employed method is based on paramagnetism. In this approach, cells take up magnetic particles^16^ and a magnetic field is then applied to aggregate cells into 3D constructs by levitation. Due to the simplicity in execution and high-throughput nature of the method, paramagnetic object manipulation (i.e., cells with attached magnetic particles) has been extensively applied in different research areas such as tumor engineering^17^ and stem cell engineering^18^ for drug testing and stem cell differentiation. Another magnetic force-based method for cell manipulation is based on the Magneto-Archimedes effect,^19^ which makes use of differences in magnetic susceptibility between a target object (usually a diamagnetic object, i.e., cells) and its surrounding paramagnetic medium made of paramagnetic agents. A paramagnetic agent like some paramagnetic salts (Gadobutrol, MnCl_2_, etc.) is applied in the presence of a magnetic field to generate magnetic discrepancies in the system, which helps levitate and assemble cells into multi-cellular spheroids with controlled geometries.^20^ As there is no need for any matrix to position cells and create complex geometries, such magnetic force-based cell manipulation methods have garnered increasing interest for 3D model reconstruction^20^ and cell density measurements.^21^

In this Review, we outline and discuss the potential of magnetic force-based cell manipulation techniques for reconstructing complex tissue constructs *in vitro* in tumor and tissue engineering applications. Furthermore, the integration of these techniques with microfluidics will also be discussed, paving the way for more complex and precise tissue engineering and cell biology research.

## II. PARAMAGNETIC OBJECT MANIPULATION

### A. Definition

Paramagnetic object manipulation, also known as positive magnetophoresis, enables the assembly of cells when an external magnetic field is applied in a diamagnetic medium such as water, generating an attractive magnetic force that directs cells into specific 3D cellular assemblies.^22–24^ As cells do not possess intrinsic magnetic properties, they are typically “labeled” with magnetic nanoparticles (MNPs) or beads. This causes them to exhibit greater magnetic susceptibility than the surrounding buffer or medium, resulting in cell movement toward the field maximum.^17^ MNPs are preferentially used due to their high biocompatibility, tunable functionality, large surface area to volume ratio, and selective binding to target cells due to the presence of highly specific recognition ligands that can be coupled onto their surface.^23,24^ The magnetic field itself can be generated in a customized manner using externally positioned rare earth magnets, which are comparatively simpler and much less expensive than the components required in other methods for cell assembly such as 3D bioprinting.^17,24^ The magnetic force ( $F_{m}$) of positive magnetophoresis is given by the following equation:^25^

$$F_{m}=\frac{X_{s}V}{\mu_{0}}\left(B\cdot \nabla \right)B,$$ (1)

where $X_{s}$ is the magnetic susceptibilities of the target substance, V is the volume of the target substance, $\mu_{0}=4\pi \times 10^{-7}\;(N\;A^{-2})$ is the magnetic permeability of a vacuum, and B is the magnetic field vector. Equation (1) shows that the magnetic force will increase if the substance is a paramagnetic or superparamagnetic one ( $X_{s}\;\text{increases}$), which will drive it to the field maximum. Paramagnetic manipulation has been widely employed in cellular and tissue engineering fields, particularly for *in vitro* cell sorting and detection of cells. For example, magnetically activated cell sorting (MACS) is a widely used technique that uses MNPs coupled with cell surface antigen-specific antibodies to bind to cells on ferromagnetic matrices in a high-gradient magnetic field. Non-MNPs binding cells are then filtered out, while magnetically tagged cells with varying degrees of magnetic susceptibility are retained and gradually eluted, allowing for cell numbers above 10^10^ to be processed quickly, while preserving high purity, cell viability, and integrity.^26^ Multiparameter MACS has been used for a variety of applications such as for the detection and isolation of rare subsets of cells [e.g., uncommitted subsets of human hematopoietic progenitor cells and circulating tumor cells (CTCs)^27^] which can be used for determining cancer prognosis and personalized chemotherapy.^28^ Furthermore, cell sorting technologies using differential magnetic bead distribution have been developed by exploiting the inherent endocytic capacity or exploiting the intrinsic magnetic properties of iron-containing erythrocytes.^24,29^ Furthermore, a combined approach integrating positive magnetophoresis and microfluidics has resulted in “magnetofluidics,” which allows for continuous flow and contactless separation of cells.^24^

Paramagnetic manipulation has also been used to assemble cells into 3D cellular spheroids, guide cells into multi-cellular sheet-like structures to optimize cellular contact, and enhance the seeding efficiency of cells into scaffolds for tissue engineering applications. In this section, we will discuss recent studies that have leveraged paramagnetic object manipulation for the generation of 3D tissue structures *in vitro*. We will also discuss how paramagnetic cell manipulation has been integrated with other techniques such as microfluidics.

### B. Applications in tissue engineering

In this section, we will specifically focus on how paramagnetic object manipulation has been used for magnetic homing of cells into 3D tissue configurations including spheroids.

**FIG. 2.**
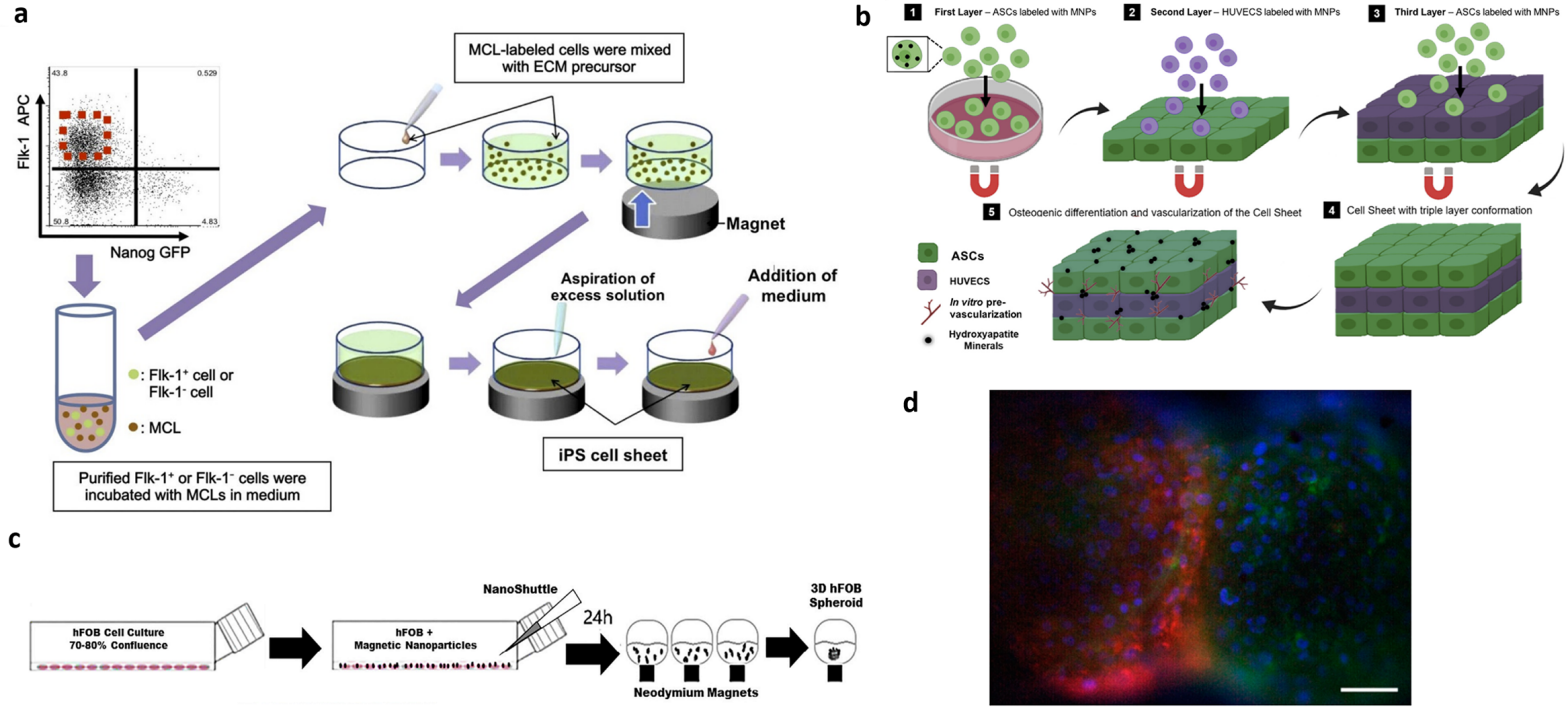
Positive magnetophoresis applications in tissue engineering. (a) Procedure for constructing iPSC-derived cell sheet. Adapted with permission from Kito *et al.*, Sci. Rep. **3**, 1418 (2013). Copyright 2013 Authors, licensed under a Creative Commons Attribution (CC BY) license.^32^ (b) Schematic representation of the fabricated 3D vascularized heterotypic cell sheet. Adapted with permission from Silva *et al.*, Biomaterials **231**,119664 (2020). Copyright 2020 Copyright Clearance Center, Inc.^33^ (c) Magnetically labeled human fetal osteoblast (hFOB) cells were seeded and guided to form an air–liquid interface and aggregated into a 3D spheroid. Adapted with permission from Gaitan-Salvatella *et al.*, Front. Mol. Biosci. **8**, 672518 (2021). Copyright 2021 Authors, licensed under a Creative Commons Attribution (CC BY) license.^38^ (d) Fluorescence image showing two merged 3T3-L1 preadipocytes spheroids after 24 h culture. Adapted with permission from Jafari *et al.*, ACS Biomater. Sci. Eng. **5**(5), 2532–2542 (2019). Copyright 2019 Copyright Clearance Center, Inc.^39^

In the absence of any scaffold, magnetically labeled cells can be manipulated to form large, multi-cellular tissue constructs. In one example, magnetically labeled human mesenchymal stem cells (MSCs) not only maintained the ability to differentiate into osteoblasts, adipocytes and chondrocytes, but these cells could be utilized to construct multi-layered sheet-like structures within a 24 h culture period, enabling bone growth and repair of small rat cranial bone defects.^30,31^ The use of an electromagnet allowed for facile harvesting of the MSC sheets, which were easily detached from the culture surface after removal of the magnetic field. Positive magnetophoresis has also been used to induce bone angiogenesis with induced pluripotent stem cells (iPSCs). Kito *et al.* proposed a co-culture of mouse iPSCs and magnetic nanoparticle-encapsulated liposomes (MCLs). By using a magnet to manipulate MCL-labeled iPSCs mixed with ECM, multi-layered cell sheets composed of magnetized iPSCs were formed. These cell sheets not only accelerated re-vascularization of ischemic hindlimbs of mice but also increased the expression of vascular endothelial growth factor (VEGF) and basic fibroblast growth factor (bFGF) in ischemic tissue, demonstrating the potential for angiogenesis therapy [Fig. 2(a)].^32^ In another example, Silva *et al.* recently generated a novel pre-vascularized multi-cellular sheet construct using adipose-derived stem cells (ADSCs) and human umbilical vein endothelial cells (HUVECs) in a triple sheet-layered conformation. This cellular assembly led to the self-generation of vital growth factors such as bone morphogenetic protein-2 (BMP-2) and VEGF, promoting both angiogenesis and osteogenesis for bone regeneration [Fig. 2(b)].^33,34^ Recently, Vinhas *et al.* developed a tendon model using magnetic cell sheets comprising human-derived tendon cells, which demonstrates promise for tendon regeneration and *ex vivo* tendon disease modeling. This model showed improved cellular organization and matrix deposition compared to conventional methods and exhibited *in vivo*-like tenogenic and immunogenic potential.^35^ This technique has been utilized in several studies to generate a wide variety of different cell sheet types, such as that comprising MSCs, ADSCs, dermal cells, human dermal fibroblasts, and human retinal pigment epithelial cells, and has also been used in the generation of complex, multi-layered, and even tubular structures.^36,37^

In addition to cell sheets, positive magnetophoresis has also been used to create 3D spheroid constructs for tissue engineering. Current methods to generate spheroids involve culturing cells under continuous stirring conditions, and confining cells using hanging drop or non-adherent configurations. However, these methods can be difficult to scale-up and may confer little control over the uniformity of spheroid formation.^38,40^ These methods are also “passive” approaches, lacking external forces that promote contact between cells or the fusion of cellular aggregates. Hence, there is limited spatiotemporal control over cell aggregation and spheroid formation.^39^ In contrast, magnetic force-based guidance is an “active” yet non-contact approach to achieve spheroid formation. This method can be applied to a larger variety of cell types and advance rapid and high-throughput assembly, while maintaining cell viability and functionality.^38^ It also confers greater control over spheroid size, sphericity, and maturation time as compared to conventional passive spheroid culture methods, while allowing for increase in complexity due to easy incorporation of multiple cell types.^40^ Positive magnetophoresis can be used to create spheroids by placing a cell culture chamber above permanent magnets, which can aggregate cells into spheroids in a multi-step seeding process. As an alternative to gold standard therapies and scaffold-based methods for bone defect repair, 3D spheroids using human fetal osteoblasts (hFOB) were created under magnetic levitation using the NANOSHUTTLE^™^ system from Greiner Bio-One company, comprising iron oxide and gold nanoparticles cross-linked with poly-L-lysine; poly-L-lysine promotes magnetic association with hFOB cell membrane [Fig. 2(c)].^16,38^ Neodymium magnets were then used to guide labeled-hFOBs into stable, compact, medium-sized 3D osteoblast spheroids; the control of spheroid size enabled adequate diffusion of oxygen and nutrients, maintaining viability, integrity, and continuous cell proliferation beyond 14 days of cell culture, as compared to two-dimensional (2D) monolayer-cultured cells. The spheroid-cultured cells also demonstrated evidence of osteogenic differentiation and exhibited *in vivo*-like cell–cell and cell–ECM interactions. Being a relatively recent development, this model could be very useful for gaining mechanistic insights into bone formation and regeneration processes, surpassing limitations of primary cell cultures that are usually obtained from normal human or rodent bone tissue.^38^

Paramagnetic manipulation has also shown significant promise in generating spheroids comprising co-cultures of different cell types. The NANOSHUTTLE^™^ was used to reconstruct 3D co-cultures representing the bronchiole, comprising an epithelial cell layer, smooth muscle cells, pulmonary fibroblasts, and endothelial cells in a sequential manner, akin to native bronchiole organization.^16,40,41^ Similarly, Tseng *et al.* used the above technology to create an aortic valve model comprising aortic valve cells, valvular interstitial, and endothelial cells in a 3D co-culture setting, allowing for the recapitulation of vasodilation and vasoconstriction, similar to *in vivo* vasoactivity.^41,42^ Importantly, this study showed the potential of these constructs for high-throughput screening, potentially overcoming existing limitations of *in vivo* assessments that require isolation of fresh tissue from animals. The above technology was also used to enable co-culture of adipocytes with endothelial cells to form a vascularized adiposphere,^43^ while another study by Fayol *et al.* utilized this method to enhance luminal endothelialization, demonstrating potential for vascular tissue engineering.^44,45^ In another system, the magnetic 3D bioassembly (M3DB) platform was used to create salivary gland (SG) organoids and hDPSC 3D cultures, enabling the generation, isolation, and identification of extracellular vesicles (EV) to investigate biological repair processes and identify novel signaling cues.^18^ This system can potentially optimize epithelial SG repair after injury caused by radiation therapy in patients with advanced head and neck cancers. Muscle tissues have also been created by guiding magnetically labeled cells using a ring-shaped magnet under the culture plate for high-throughput *in vitro* vasoactivity assay development.^42,46,47^ Different geometries have also been explored; Whatley *et al.* developed a quick and precise magnetic-based technique, in which they utilized an external magnetic field to direct superparamagnetic iron oxide nanoparticles (SPIONs)-labeled endothelial cells into spheroids. These structures were then guided into specific positions on CAD-printed structures, demonstrating the potential for scalable fabrication of complex 3D multicellular tissue structures consisting of various cell types and prefabricated magnetic patterns. This approach may have use in bioprinting and multi-cellular tissue graft fabrication.^48^ Going beyond simple spheroid structures, O'Connor *et al.* modulated the patterns of magnetic fields to tune shapes of cell assemblies. First, they fabricated uniform and stable cellular spheroids using 3T3-L1 preadipocytes and permanent magnets placed under cell culture plates, and then converted them to hybrid multicellular aggregates by co-culturing with magnetically labeled fibroblasts. They also used an external magnetic field to restrict tissue remodeling and retain desired tissue structures over time in the cultures. They then fabricated ring-like spheroids and tried to merge two spheroids into one by using positive magnetophoresis [Fig. 2(d)].^39^ This example illustrates the possibility of generating and controlling complex tissue architectures using magnetic forces which may be important for modeling after spatially heterogeneous tumors.

Magnetoferritin (MFt) is a biological MNP that not only provides magnetic functionality but also preserves cell viability. Ferritin, a key component in MFt, can be categorized into two types based on its distribution in the human body, intracellular and extracellular.^49^ Predominantly found in the cytosol, ferritin also exists, albeit in smaller quantities, in the nucleus and in a different form in the mitochondria of most tissues. Ferritin plays a crucial role in iron storage and maintaining iron balance in the body.^50^ It protects both mitochondria and DNA from oxidative harm and iron toxicity. Moreover, the heavy subunit of ferritin is involved in a myriad of biological processes, including cell proliferation, angiogenesis, and the expansion of stem cells.^51^ MFt, a novel magnetic nanomaterial, is synthesized via biomimetic mineralization, which involves the formation of magnetic iron oxide within the cavity of ferritin. Noted for its excellent biocompatibility and flexibility, MFt is highly suitable for various biomedical applications and is often referred to as a superparamagnetic protein.^52^ Such MNPs have been used to create a Janus structure of magnetic cellular spheroids by incorporating MNPs in the ECM and inducing the formation of spheroids via the hanging drop method.^53^ Furthermore, cell surface engineering [e.g., through the deposition of poly(allylamine)] on MNPs has been shown to preserve cell viability by reducing the probability of cytoplasm penetration, maintaining membrane integrity and essential cellular functions.^54^ Additionally, cells can be manipulated via positive magnetophoresis by incubating cells in a substrate with iron oxide-encapsulated polymeric nanoparticles or by attaching cells to magnetic collagen/cell-bead surfaces to be manipulated within an externally created magnetic field.^55^ Koudan *et al.* developed a novel polymeric capsule that encapsulated iron oxide nanoparticles for magnetic bioassembly of magnetically labeled murine fibroblast cells; this approach reduces the toxicity of MNPs as they are not in direct contact with cellular components and oxidative sites, and hence reduce DNA damage. This enhances cell integration and retention for extended periods of time and significantly improves magnetic properties due to the coherence effect of putting hundreds of nanoparticles into one entity.^56^ Hence, this model could be applied for tissue spheroid patterning, magnetic bioprinting and tissue spheroid characterization.

In sum, the compatibility of positive magnetophoresis with a wide range of cell types in the creation of different tissue spheroidal constructs highlights the versatility of this approach for many tissue engineering applications.

### C. Applications in tumor modeling

Two-dimensional (2D) cancer monolayer cultures have been the model of choice for drug development and cancer biology studies due to their ease of use, low cost, and reproducibility.^39,40^ However, monolayer cultures poorly recapitulate key *in vivo* features such as cellular heterogeneity, cell–cell signaling, and cell–ECM interactions. Accordingly, these models recapitulate to a very limited extent, tumor growth kinetics, drug kinetics, protein expression, and biomolecule distribution.^57^ Unlike 2D cultures, spheroid cultures are better able to recapitulate the *in vivo* 3D architecture, physical, chemical, and biological gradients, as well as cell–cell and cell–ECM interactions in tumors. Additionally, the incorporation of different cell populations such as fibroblasts and endothelial cells with cancer cells allows for the mimicking of cellular heterogeneity and tissue complexity.^39,40,57^ Accordingly, spheroid models are now widely used for mechanistic studies, drug screening, and personalized drug testing.^58,59^

**FIG. 3.**
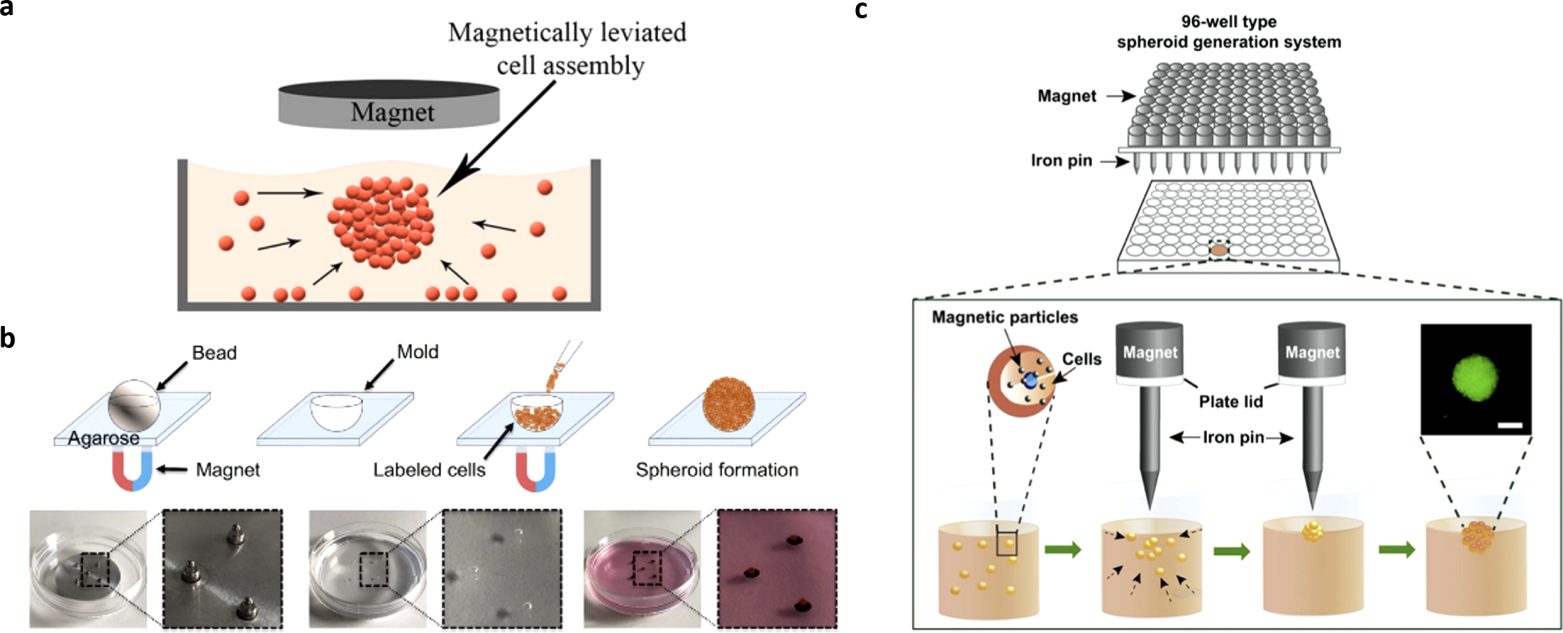
Positive magnetophoresis applications in tumor modeling. (a) 3D cell culture with magnetic-based levitation. Adapted with permission from Yaman *et al.*, Front. Bioeng. Biotechnol. **6**, 192 (2018). Copyright 2018 Authors, licensed under a Creative Commons Attribution (CC BY) license.^24^ (b) Magnetically labeled cells are seeded and aggregated into 3D spheroids using an externally applied magnetic force. Adapted with permission from Perez *et al.*, Biofabrication **13**(1), 015018 (2020). Copyright 2020 Authors, licensed under a Creative Commons Attribution (CC BY) license.^17^ (c) Schematic showing spheroid generation platform using magnetic nanoparticles and iron pins. Adapted with permission from Kim *et al.*, Biomaterials **34**(34), 8555–8563 (2013). Copyright 2013 Copyright Clearance Center, Inc.^64^

Souza *et al.* used a positive magnetophoresis-based method composed of magnetic MNPs and filamentous bacteriophages to assemble human glioblastoma and astrocyte cells into specific geometries and enable co-culture multi-cellular assembly [Fig. 3(a)].^16,24^ Subsequently, Souza *et al.* advanced their technology by developing a bacteriophage-free, nontoxic, noninfectious, and non-inflammatory hydrogel, commercialized as NANOSHUTTLE^™^, for the formation of cellular spheroids of different 3D geometries within a physiologically relevant ECM.^60^ This MNP technology was used to develop a large-size 3D co-culture model that mimics the heterogeneous microenvironment of breast tumors for anti-cancer drug testing.^60,61^ Proteomic studies on protein regulation and abundance in cancer cell ECM can potentially also be evaluated using the NANOSHUTTLE™ system.^62^ Enhanced understanding of ECM regulation, its role in tissue homeostasis, and cancer pathogenesis have the potential to support drug testing efforts. Significantly, the NANOSHUTTLE™ system was used in the development of a first-in-class automated, robotic 3D bioprinting with magnetic incubator shelf assemblies for quick assembly of a large number of tissue spheroids.^63^ This technology has the potential to advance personalized medicine by allowing rapid assembly of tissue spheroids from patient-derived cells, which can be utilized for high-throughput drug testing and screening. Perez *et al.* used the above technology to set up a highly tunable 3D tumor spheroid model with tightly controlled sphericity using two different cell lines (murine colon carcinoma cells and human glioblastoma cells), obtaining mature spheroids within 24 h of incubation [Fig. 3(b)].^17^ Reproducibility and the ability for scaling up to accommodate different cell lines capable of magnetic nanoparticle internalization was demonstrated. Increased accuracy and control over spheroid size was demonstrated by Kim *et al.*, in which a magnetic pin-array system comprising a combination of magnets and iron pins was used to generate a strong and highly focused magnetic field at a specific location. The resulting cell–cell contacts allowed for tight assembly of MNP-incorporated cells into a 3D spheroid [Fig. 3(c)].^64^ In comparison to the conventional hanging drop method, Tang *et al.*, developed a high-quality multicellular tumor spheroid (MCTS) induction platform based on an anisotropic magnetic hydrogel. The MCTS platform supported lower cell apoptosis and better cancer cell viability, demonstrating its potential as an *in vitro* platform for tumor spheroid formation and drug efficacy evaluation.^65^ Ho *et al.* developed a multi-cellular spheroid model with magnetically labeled human cervical adenocarcinoma cells, which showed good cell viability and F-actin distribution similar to 3D organization of cellular cytoskeleton. The application of a magnetic field allowed for easy and quick separation of spheroids without the need for centrifugation, while allowing for precise positioning of spheroids to form a larger tissue construct.^66^

### D. Integration of paramagnetic cell manipulation with microfluidics

In recent years, paramagnetic object manipulation has also been combined with microfluidics in an emerging approach called magnetofluidics. In magnetofluidics, an external magnetic force can be applied with a simple magnet, which then easily manipulates labeled particles, such as cells inside microfluidic channels, in a non-contact manner and under continuous flow.^24^ As magnetofluidics allows for more precise control through manipulation of size, shape, magnetization, flow velocity, channel dimensions, and fluid properties, this method has the potential for high-throughput fluidic processes in a variety of tissue engineering applications.^38^ Magnetofluidic systems based on positive magnetophoresis has been used in mixing, separation, and sorting of cells, particularly in the isolation of rare cells such as CTCs. The reader is directed to reviews by Nguyen *et al.* and Yaman *et al.*, which cover the various applications of magnetofluidics in *in vitro* drug testing, labeling of magnetic beads, and specific binding of target molecules. Efficient mixing, separation, and detection on a microfluidic lab-on-chip device (LOC) allows for the development of a fully automated system, enabling high-throughput applications. Trapping of cells is also enabled through microfluidics, which can later be used for further culture, drug testing, and assembly into tissues. Utilizing an external magnetic field can trap and concentrate cells of specific subtypes to investigate cell–cell interactions, regulation mechanisms, and multi-cellular assembly through better recapitulation of physiological environments. Many applications show more than 80% trapping and mixing efficiency, with minimal sample loss, increased accuracy, and reduced time and cost.^23^ Furthermore, as sample flow is laminar in most microfluidic applications, the application of an external magnetic field ensures that particles gain enough energy to overcome existing viscous forces. However, a possible complication in using positive magnetophoresis is the limited particle size, due to aggregation of large particles, especially under high concentration, in microfluidic channels which might clog the flow path and make mixing more difficult.

### E. Limitations and design strategies for paramagnetic cell manipulation

Paramagnetic object manipulation surpasses existing limitations in conventional methods of cellular assembly such as hanging drop, spinner culture, and rotating cell culture, by enabling high-throughput production of tissue constructs while being relatively less expensive, labor intensive, and time-consuming.^24^ They also ensure a greater degree of control and manipulation flexibility over the size and uniformity of 3D spheroids and tissue constructs of defined geometries in 2D and 3D cell cultures, while allowing cells with extremely small magnetic susceptibility to be efficiently assembled.^26^ However, magnetic labeling can also be time-consuming, manually intensive, and prone to experimental variability due to magnetic moment variation and cell labeling efficiency. Moreover, the cell compatibility of paramagnetic object manipulation is influenced by many factors. It is limited by issues such as inadequate cellular internalization of MNPs and potential biological interference of MNP labels, which could affect the standardization of experimental procedures.^24,25,67^ However, this could potentially be resolved through emerging automatic technologies such as the magnetic 3D bioassembly (M3DB).^24^ Furthermore, long-term retention of MNPs and prolonged exposure to powerful magnetic fields may affect cellular function and viability of highly sensitive cell types such as stem cells and progenitor cells.^68^ Moreover, internalization of micro- and nano-particles could cause the generation of reactive oxygen species (ROS), damaging the structure and function of cellular components.^69,70^ Moreover, MNPs internalization may also affect cell nuclear activities and result in leaky cell membranes, which may further cause abnormal cell metabolism or even cell death.^71^ However, this could be potentially be resolved through the encapsulation of MNPs in polymer or composite capsules as demonstrated by Koudan *et al.*^56^ Further research is required for establishing optimized techniques through fine-tuning various parameters to enhance multi-functionality when dealing with multiple cell types or rare cells such as CTCs, which show significant genetic and phenotypic heterogeneity.^60^

To design an optimized paramagnetic manipulation system with good cell compatibility, several factors need to be considered. First, the size of MNPs needs to be optimized depending on the cell characteristics. As cells poorly internalize larger MNPs, the only way is to leverage ligand-receptor binding interactions, which is time-consuming due to the need for surface modifications on MNPs. Lacerda *et al.* reported^72^ that larger magnetic nanoparticles (MNPs) tend to attract a greater quantity of proteins, which can, in turn, reduce their cooperativity. This is primarily due to the intensification of protein packing within the MNPs. This may trigger the loss of protein function resulting in abnormal cellular activities.^72^ Smaller MNPs are more easily internalized by cells.^73^ However, too much MNP internalization may cause the aggregation of MNPs within cells. The metallic nature of metal-based NPs and transition metals generate ROS, which may lead to cell apoptosis.^69^ Second, surface-modified MNPs may aid in making MNPs more biocompatible in different applications. Molecules like dextran and phospholipids are commonly used for the surface modification of MNPs which can not only bridge MNPs and cells but also prevent MNPs from causing oxidative stress or other types of cellular damage.^74^ Third, magnetic properties also play a role. The magnetic properties of MNPs need to be strong enough to control cells but not to the extent of causing cellular dysfunction. For example, SPIONs are often used because they can be manipulated with an external magnetic field but do not retain magnetism when the field is removed, reducing the risk of unwanted magnetic effects on cells.^75^ Finally, the clearance method taken by cells to remove MNPs should also be taken into consideration. After internalization, there are two potential routes for cells to clear MNPs. The first one is endocytic pathway and lysosomal degradation.^76^ Once internalized, MNPs often end up in the endosomes and then are transported to lysosomes where they can be degraded by acidic pH and various enzymes. However, depending on their composition, some MNPs may be resistant to this degradation. The second method is exocytosis.^76^ This is an ATP-consuming process where cells release some vesicles encapsulating MNPs. If these two methods fail to remove MNPs, MNPs will remain with cells for a long period and be distributed to daughter cells during cell division, which may finally result in cell death.^77^ Other factors such as the concentration of MNPs are also critical to consider since high concentrations of MNPs may cause apoptosis.^78^

## III. DIAMAGNETIC OBJECT MANIPULATION

### A. Definition

In contrast to paramagnetic object manipulation, diamagnetic object manipulation, commonly known as negative magnetophoresis, manipulates diamagnetic or weakly paramagnetic objects by using the Magneto-Archimedes effect. The Magneto-Archimedes effect takes advantage of magnetic differences between a target material and its surrounding paramagnetic medium for the separation, assembly, and levitation of cells. The magnetic force (*F_m_*) of negative magnetophoresis^79^ is given by the following equation:

$$F_{m}=\frac{\left(X_{s}-X_{m}\right)V}{\mu_{0}}\left(B\cdot \nabla \right)B,$$ (2)

where $X_{s}$ and $X_{m}$ are the magnetic susceptibilities of the target substance and surrounding medium, respectively, V is the volume of the target substance, $\mu_{0}=4\pi \times 10^{-7}\;(N\;A^{-2})$ is the magnetic permeability of a vacuum, and B is the magnetic field vector. Equation (2) shows that the target substance, such as a diamagnetic or paramagnetic object with much lower magnetic susceptibility than that of the surrounding medium, enables this type of manipulation. In other words, if a target substance were to be placed in a paramagnetic medium, it can potentially be maneuvered from a high-field to low-field region based on diamagnetism, without any labeling of the target substance needed. This is because an inhomogeneous magnetic field imposed externally will drive the surrounding paramagnetic medium to the field maximum, forcing the target substance to the field minimum. This effect can be amplified by increasing the magnetic field gradient or the magnetic susceptibility of the surrounding medium (typically a paramagnetic salt solution or ferrofluid).^19,80^ Compared to Eq. (1), Eq. (2) includes the magnetic susceptibility of the surrounding medium, $X_{m}$, which keeps the value of $\left(X_{s}-X_{m}\right)$ in a low range since the target substances are typically diamagnetic objects with lower magnetic susceptibility than the surrounding medium.

**FIG. 4.**
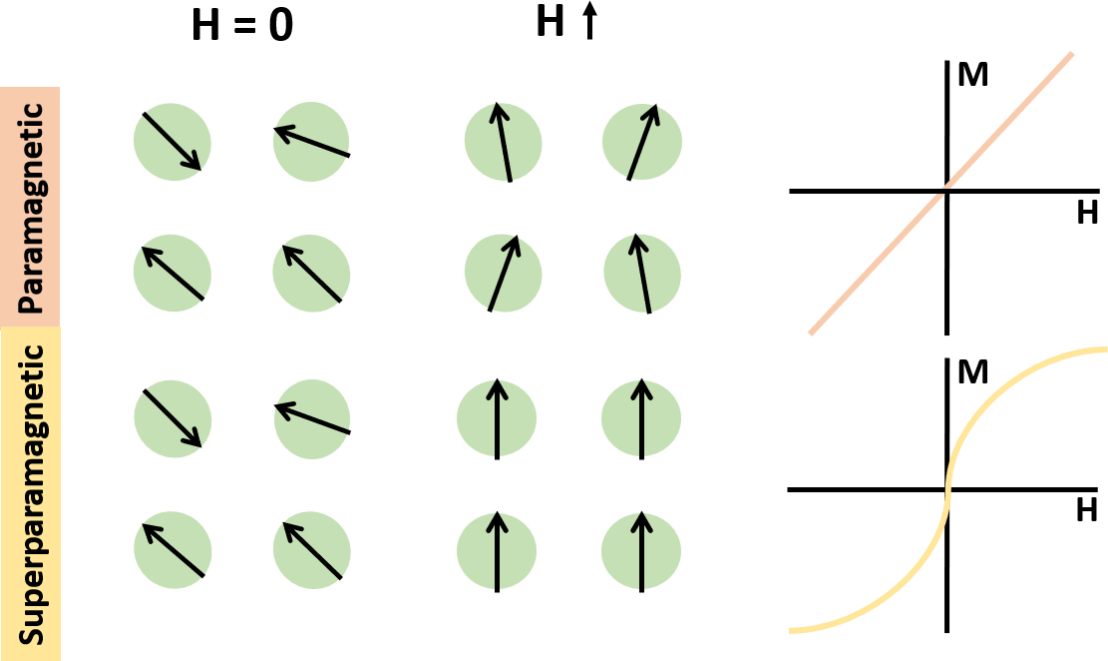
Comparison between paramagnetic and superparamagnetic substances regarding the relationship of magnetic moment *M* with the external magnetic field *H* and *M*–*H* curve. Superparamagnetic substances enable a faster response orderly under an external magnetic field.

The critical factor within the paramagnetic medium which induces movement of the target substance is the paramagnetic agent. Due to differences in magnetic susceptibilities, paramagnetic agents are typically divided into two groups, paramagnetic and superparamagnetic substances. Manganese (II) chloride (MnCl_2_)^81^ and Gd^3+^ chelates (Gadolinium,^20,82^ Gadabutrol^83^ etc.) are two of the most commonly used paramagnetic agents for diamagnetic objects manipulation with magnetic susceptibilities in the range of $10^{-3}$ (dimensionless),^84^ while SPIONs attain two orders of larger magnetic susceptibilities (Fig. 4).^85^

### B. Applications in spheroid assembly

**FIG. 5.**
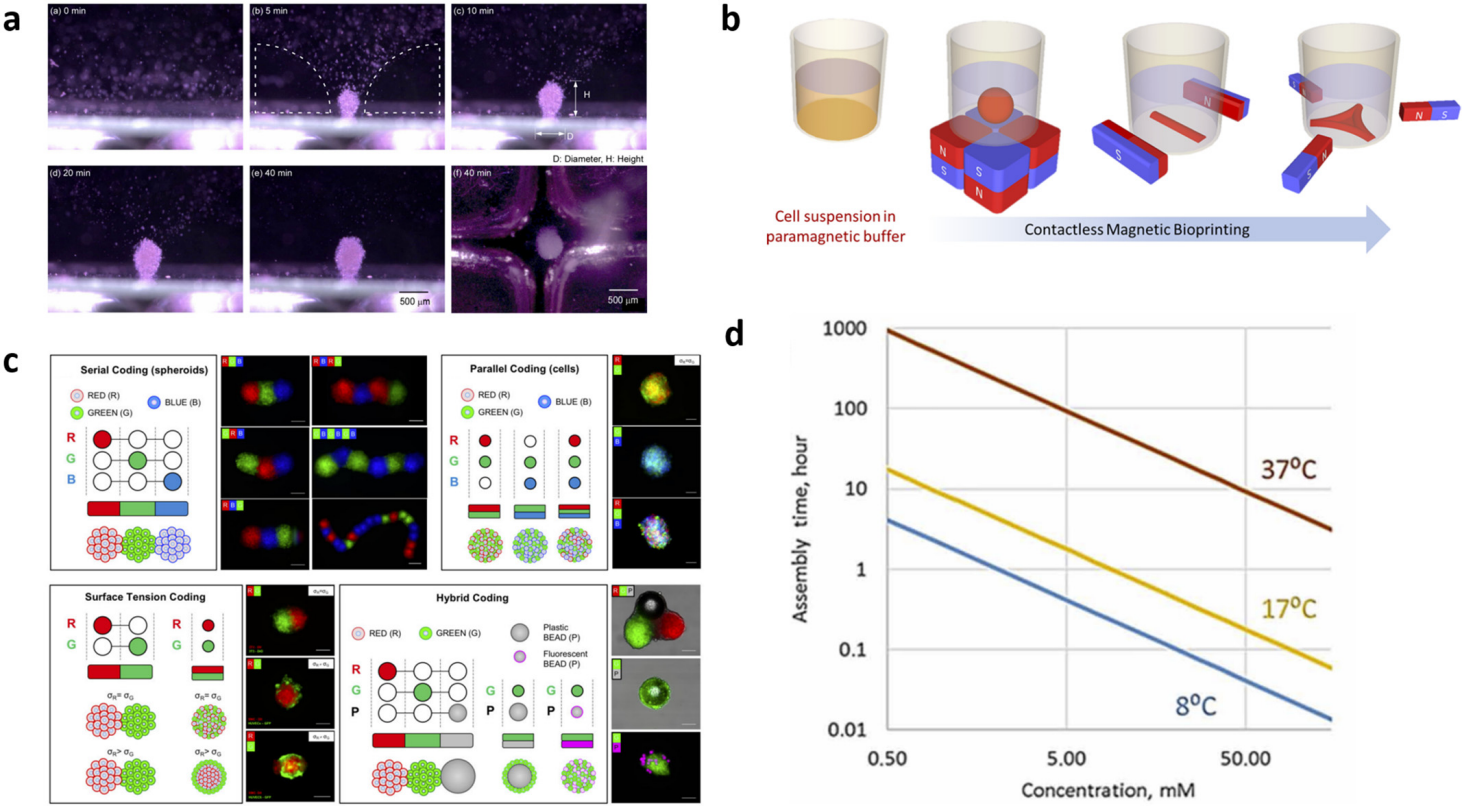
Negative magnetophoresis applications in spheroid cultures. (a) Sequential images of spheroid formation as viewed from the side during 40 min. Reproduced with permission from Akiyama and Morishima, Appl. Phys. Lett. **98**(16), 163702 (2011).^92^ Copyright 2011 AIP Publishing. (b) A novel macroscale, contactless, label-free method to print *in situ* three-dimensional (3D) cell assemblies of different morphologies and sizes. Reproduced with permission from Abdel Fattah *et al.*, ACS Biomater. Sci. Eng. **2**(12), 2133–2138 (2016). Copyright 2016 American Chemical Society.^88^ (c) Coding of spatially controlled cellular architectures with heterogeneous compositions. Adapted with permission from Tocchio *et al.*, Adv. Mater. **30**(4), 1705034 (2018). Copyright 2018 Copyright Clearance Center, Inc.^20^ (d) Kinetics of the construct assembly as a function of paramagnetic salt concentrations and temperature. Adapted with permission from Parfenov *et al.*, Sci. Adv. **6**, eaba4174 (2020). Copyright 2020 Authors, licensed under a Creative Commons Attribution (CC BY) license.^90^

Diamagnetic object manipulation has been tested as an approach to reconstruct tissues in tissue engineering. By using negative magnetophoresis, cells are able to self-assemble to generate cellular aggregates. In 2011, Morishima *et al.* applied four cubic magnets with opposite poles next to each other to generate an inclined magnetic gradient from the periphery to center. Combined with gadolinium added to the cell culture medium, this simple device enabled aggregation of HH cell line into a suspended spheroid in a plastic tube at the field minimum. After 3 days, cells in the spheroid started to proliferate even in the presence of low concentrations of gadolinium (<8.7 mM). This study demonstrated the potential of using the Magneto-Archimedes effect to achieve label-free spheroid formation [Fig. 5(a)].^86^ In the same year, this group demonstrated the feasibility of using this phenomenon to achieve higher throughput spheroid formation. Using the same setup of magnet arrays, the authors added more magnets to attain more spots with minimum magnetic flux densities, enabling the simultaneous formation of multiple spheroids.^86^ In 2012, this group was the first to integrate the Magneto-Archimedes effect with microfluidics.^87^ Six cellular aggregates were formed in a microfluidics chip at the same time under perfusion.

Beyond simple spheroid assemblies, more recent studies investigated the use of Magneto-Archimedes effect to create complex tissue architectures. In 2016, Puri *et al.* were the first to propose the concept of magnetic bioprinting and printed different *in situ* patterns using negative magnetophoresis.^88^ Suspended whole blood cells could be easily assembled into various cell geometries using different magnet configurations. After dispersing cells into a paramagnetic medium under an external magnetic field, due to cell magnetophoresis, the cell culture medium started to recirculate and push cells to the magnetic field minimum. Using this phenomenon, the authors demonstrated how tissue geometry can potentially be programmable in a label-free manner. Sphere, line, and three-pointed star structures were formed by using different configurations of magnets [Fig. 5(b)].^88^ In 2018, Demirci *et al.*^20^ developed a new magnetic levitation system composed of magnets, glass capillary, and mirrors. This system enabled the fabrication of living materials, specifically cells in this case, with controllable geometries and real time monitoring and imaging. Tumor cell lines, specifically NIH 3T3 cells, were assembled into tumor spheroids and encapsulated in fibrin gel derived from ECM, which enabled the formation of cell–matrix interactions. This is a novel approach to reconstruct an *in vivo* like microenvironment using tumor spheroids assembled using paramagnetic manipulation. The tumor spheroids exhibited high viability after several days of culture and invasive behavior as observed from gel degradation caused by matrix metalloproteinases (MMPs) produced from tumor cells. Due to diamagnetic tendency, they successfully merged several spheroids into one at the field minimum, showing the potential for the interaction of biological units such as single cells to spheroids or spheroids to spheroids [Fig. 5(c)]. By using this phenomenon, the tumor immune-contexture can potentially be reconstructed to study immune suppression, particularly the limited infiltration of T cells into tumor tissues.^7^

In 2018, Demirci and Mironov *et al.*^82^ fabricated a label-free, negative magnetophoresis-based prototype device made of annular magnets and glass cuvettes in the presence of gadolinium added to cell culture medium to reconstruct chondrospheres, extrapolating their fusion technology to regenerative medicine. Fused chondrospheres were found to be capable of attaching and spreading on damaged cartilage surfaces and have therapeutic effect on cartilage lesions.^89^ In 2020, Demirci and Mironov *et al.*^90^ tested their platform technology in space with microgravity, where they demonstrated for the first time, spheroid assembly and fusion of dispersed cells using the Magneto-Archimedes effect. Interestingly, this group also used simulation data to demonstrate the negative correlation between temperature, paramagnetic salt concentrations, and assembly duration [Fig. 5(d)]. Moreover, in 2020, Mauck *et al.*^91^ innovatively applied the water-absorbing quality of hydrogel to create a paramagnetic environment for diamagnetic objects like polystyrene beads, drug delivery microcapsules, and living cells which can be patterned inside the hydrogel; after cross-linking of the hydrogel, diamagnetic objects are positionally stable within the hydrogel and the magnetic contrast agent diffuses out of the hydrogel, enabling good long-term viability. They used this paramagnetic hydrogel to fabricate cartilage constructs *in vitro*. This study opened up a new avenue to use diamagnetic manipulation for tissue reconstruction.

### C. Applications in non-living object assembly

**FIG. 6.**
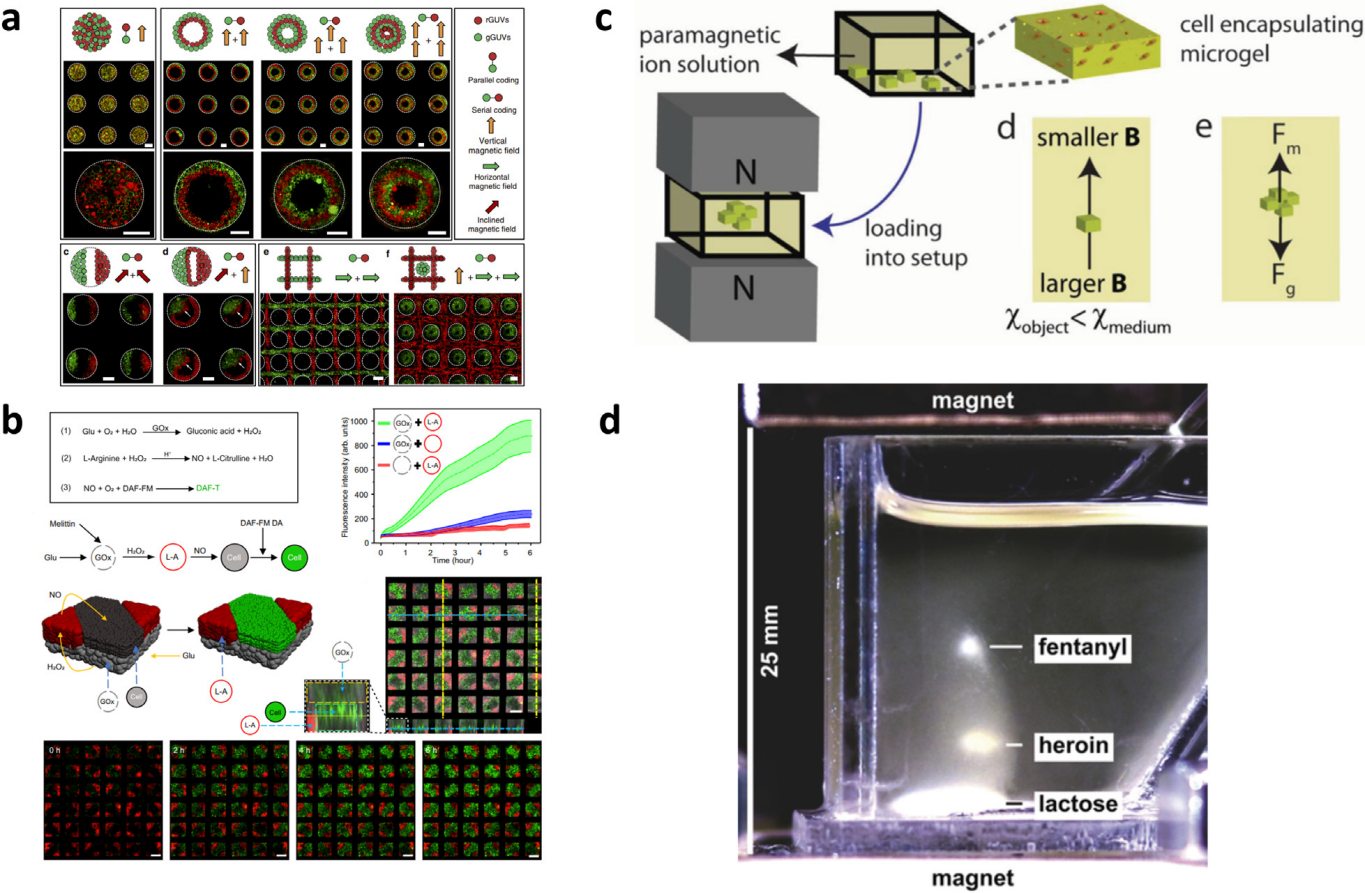
Other negative magnetophoresis applications. (a) Schematic and fluorescence images of vesicles colonies formed via the parallel coding. Adapted with permission from Li *et al.*, Nat. Commun. **11**(1), 232 (2020). Copyright 2020 Authors, licensed under a Creative Commons Attribution (CC BY) license;^81^(b) Signal communications in hybrid three-component prototissues composed vesicles and C6 glioma cells. Adapted with permission from Zhang *et al.,* Nat. Commun. 13(1), 2148 (2022). Copyright 2022 Authors, licensed under a Creative Commons Attribution (CC BY) license;^94^(c) Levitational self-assembly of cell-encapsulating building blocks in a magnetic setup composed of two NdFeB magnets with same poles facing each other. Adapted with permission from Tasoglu *et al.*, Adv. Healthcare Mater. **4**(10), 1469–1476(2015). Copyright 2015 Copyright Clearance Center, Inc.^95^(d) Successful separation, presumptive identification, and confirmatory identification of dilute fentanyl, in a mixture of heroin and a diluent (lactose). Adapted with permission from Abrahamsson *et al.*, Angew. Chem., Int. Ed. **59**(2), 874–881 (2020). Copyright 2020 Copyright Clearance Center, Inc.^98^

Beyond simple spheroid formation, negative magnetophoresis has also been used to assemble complex tissues. In 2013, Grzybowski *et al.*^93^ demonstrated the possibility of assembling colloidal particles in a nickel grid array using the Magneto-Archimedes effect. A magnetized metal device was used to generate an intrinsic magnetic field to assemble particles into spheroid-like structures. Interestingly, the authors found that particle size was an important factor that dictated movement, where colloidal particles with larger sizes tended to move to the center of the hole within the nickel grid, which is the field maximum. Following this work, in 2020, Han *et al.*^81^ leveraged magnetized stainless-steel meshes to assemble vesicles into different geometries [Fig. 6(a)]. The stainless-steel mesh was positioned at different spots of the magnet to generate heterogeneous magnetic fields. By using this mesh, the authors showed the feasibility of layer-by-layer assembly of vesicles with different geometries and the spatialized cascade of reactions between vesicles. Importantly, going beyond non-living structures, the Han group recently showed how the same platform could be used to fabricate proto-tissues instead of vesicles. These proto-tissues enabled nitric oxide production which led to the vasodilation of rat blood vessels when exposed to glucose and hydroxyurea [Fig. 6(b)].^94^

In 2015, Demirci *et al.*^95^ used negative magnetophoresis to assemble cell-encapsulating microgels into microstructures with specific patterning. First, they used photolithography with patterned masks to fabricate the microgel unit. Then, negative magnetophoresis was applied to assemble these microgel units into different geometries. Since gravity and magnetic force on the microgels were equivalent, the microgels could levitate at equilibrium state in the paramagnetic medium. When reverse assembling into nested models with variable patterning, aggregates formed from varying numbers of microgel units demonstrated different levitational heights [Fig. 6(c)]. Interestingly, other than cell or microgel assembly, Demirci *et al.* also extended their magnetic levitation system to other applications, such as cell sorting^96^ and infected cell profiling^97^ etc. Apart from living materials, the Whitesides group^98^ have used Magneto-Archimedes levitation to separate powders of illicit drugs (e.g., cocaine, methamphetamine, heroin, etc.). The device, composed of cuvette and two like-poles-facing magnets, successfully identified fentanyl in a fentanyl-laced heroin sample which was further validated by Fourier transform infrared spectroscopy-attenuated total reflectance (FTIR-ATR). Their research demonstrates the untapped potential of negative magnetophoresis coupled with analytical tools for the identification of unknown powders in mixtures [Fig. 6(d)].

### D. Limitations and design strategies for diamagnetic cell manipulation

A key limitation of using the Magneto-Archimedes effect to assemble cells is the potential cytotoxicity associated with paramagnetic agents, which is a key component to generate a repulsive force to drive cells into the field minimum. Although Gd^3+^ chelates are already used as contrast agents in magnetic resonance imaging and are suitable for use in various biosystems^20,96,99^ with high biocompatibility, high concentrations of Gd^3+^ chelates can increase the acidity of cell culture media which can negatively impact cell viability.^100^ Arslan-Yildiz *et al.*^83^ tested three types of Gd^3+^ chelates at different concentrations to investigate the effect of Gd^3+^ on cell viability and spheroid assembly. Gadobutrol was found to be the best paramagnetic agent for maintaining high cell viability in a 7-day duration. Moreover, the high toxicity of Gd^3+^ was observed at concentrations above 100 mM, in which cells stopped proliferating on the first day.

To optimize the outcome for diamagnetic cell manipulation, the choice of paramagnetic agents used and design of magnetic field are both important. As described above, it would be best to use Gadobutrol at limited concentrations (<100 mM). However, Gadobutrol has been reported to be uptaken by passive diffusion in cells with altered or compromised plasma membrane.^101^ Another study which showed the gadobutrol retention in neuronal tissues also confirmed this cellular uptake.^102^ Hence, to ensure the efficacy and compatibility of using negative magnetophoresis for cellular assembly, it is crucial to carefully evaluate the impact of paramagnetic agent uptake on cellular functions such as viability, proliferation, differentiation, and gene expression. Compared to MNPs-driven paramagnetic object manipulation, several indirect methods can be used for diamagnetic object manipulation. For example, magnetized metal plates with holes can generate magnetic gradients across the hole to effect spatial assembly of cells within each hole.^81^ Future explorations in this field could include alternative designs to further broaden the applications of diamagnetic cell manipulation. For instance, a magnetized metal plate featuring a rectangular aperture could be employed to create structures using endothelial cells, thus facilitating the generation of vessel-like structures. Such an approach could usher in a new era of customizable biological modules specifically designed to meet a broad array of biomedical needs. Overall, the field of diamagnetic cell manipulation is poised for significant expansion. Its inherent ability to maintain high cell viability combined with its adaptability for custom-designed applications presents significant promise in tissue engineering.

## IV. DISCUSSION AND FUTURE PERSPECTIVES

Magnetophoresis is the movement of particles or living materials in an external magnetic field. It utilizes the magnetic discrepancies between target substances and surrounding medium to enable object manipulation. Although positive magnetophoresis presents drawbacks such as inadequate cellular internalization of MNPs and potential biological interference by MNPs, it is easy-to-use and scaffold-free. At present, customized magnetic plates are available commercially, which makes it easier to leverage paramagnetic object manipulation for tissue engineering. Furthermore, paramagnetic object manipulation can now be realized in multi-well plate format by integrating magnetic plates into them. Regarding the biocompatibility of MNPs, MNPs have been modified to exhibit high biocompatibility with low cytotoxicity, such as NanoShuttle-PL.^103^ In sum, although significant advances have been made in paramagnetic object manipulation, future efforts should be directed toward understanding the effect of MNP metabolism in different cell types, as well as combine the approach with other analytical tools or high-throughput devices such as flow cytometers and microfluidic systems. This may allow for the simultaneous formation of a larger number of spheroids or organoids for *in situ* characterization and detection.

For negative magnetophoresis, more efforts should be directed at maintaining cell viability by optimizing the concentration of paramagnetic agents used or using a new alternative medium with low cytotoxicity. Their direct impact on cellular metabolism has not been extensively studied and would depend on numerous factors such as the specific type of salt used, its concentration, exposure duration, and the type of cells being examined.^104^ Cells typically maintain strict regulation of their internal ion concentrations, and introducing any form of salt could potentially disrupt these concentrations and thus affect cellular metabolism.^105,106^ Paramagnetic salts could also theoretically induce oxidative stress,^107^ which could further impact metabolic processes. Hence, cellular activities like metabolism should also be better characterized to ensure cells maintain their original phenotype. Although a few groups have attempted to combine this technology with microfluidics for cell sorting^97^ or spheroid formation,^20^ further work needs to be^81,94^ carried out to demonstrate the potential of negative magnetophoresis for programmable cell assembly and compare the outcomes to 3D bioprinting. These advances will pave the way for the adoption of effective and efficient magnetic-printing methods in tissue engineering, especially complex tissue model reconstruction.

Compared to positive magnetophoresis, the development of negative magnetophoresis appears to be lagging behind as more parameters (e.g., magnetic susceptibility and effective concentration of surrounding medium, or the magnetism setup) need to be taken into consideration to achieve the intended outcome. Consequently, the use of positive magnetophoresis has been the preferred approach for assembling cells into spheroids. However, depending on the cell type, negative magnetophoresis may be a better option for extremely MNP-sensitive cells. Additionally, if negative magnetophoresis were to be further developed to enable customized and portable instruments with standardized protocols (including paramagnetic salt-related parameters) to be brought to the user, this method may also be a better alternative since it is noninvasive and FDA-approved paramagnetic salts (such as Gadobutrol) are already commercially available.^108^

We anticipate that inexpensive and portable magnetic devices, coupled with microfluidics or other techniques, will play a vital role to advance the fields of tissue engineering and tumor engineering. Moreover, the potential tandem use of these two magnetic force-based manipulations may also be interesting to explore. In the future, this may also provide insight into controlling complex tissue or tumor reconstruction by assembling different geometries within one object using different mechanisms.

## Data Availability

Data sharing is not applicable to this article as no new data were created or analyzed in this study.
